# Collagen Protein Ingestion during Recovery from Exercise Does Not Increase Muscle Connective Protein Synthesis Rates

**DOI:** 10.1249/MSS.0000000000003214

**Published:** 2023-05-19

**Authors:** THORBEN AUSSIEKER, LUUK HILKENS, ANDREW M. HOLWERDA, CAS J. FUCHS, LISANNE H. P. HOUBEN, JOAN M. SENDEN, JAN-WILLEM VAN DIJK, TIM SNIJDERS, LUC J. C. VAN LOON

**Affiliations:** 1 Department of Human Biology, School of Nutrition and Translational Research in Metabolism (NUTRIM), Maastricht University Medical Centre^+^, Maastricht, THE NETHERLANDS; 2 School of Sport and Exercise, HAN University of Applied Sciences, Nijmegen, THE NETHERLANDS

**Keywords:** WHEY PROTEIN, MYOFIBRILLAR PROTEIN, CONNECTIVE TISSUE, RESISTANCE EXERCISE, BONE MARKERS, BARBELL SQUATS

## Abstract

**Introduction:**

Protein ingestion during recovery from exercise has been reported to augment myofibrillar protein synthesis rates, without increasing muscle connective protein synthesis rates. It has been suggested that collagen protein may be effective in stimulating muscle connective protein synthesis. The present study assessed the capacity of both whey and collagen protein ingestion to stimulate postexercise myofibrillar and muscle connective protein synthesis rates.

**Methods:**

In a randomized, double-blind, parallel design, 45 young male (*n* = 30) and female (*n* = 15) recreational athletes (age, 25 ± 4 yr; body mass index, 24.1 ± 2.0 kg·m^−2^) were selected to receive primed continuous intravenous infusions with l-[ring-^13^C_6_]-phenylalanine and l-[3,5-^2^H_2_]-tyrosine. After a single session of resistance type exercise, subjects were randomly allocated to one of three groups ingesting either 30 g whey protein (WHEY, *n* = 15), 30 g collagen protein (COLL, *n* = 15) or a noncaloric placebo (PLA, *n* = 15). Blood and muscle biopsy samples were collected over a subsequent 5-h recovery period to assess both myofibrillar and muscle connective protein synthesis rates.

**Results:**

Protein ingestion increased circulating plasma amino acid concentrations (*P* < 0.05). The postprandial rise in plasma leucine and essential amino acid concentrations was greater in WHEY compared with COLL, whereas plasma glycine and proline concentrations increased more in COLL compared with WHEY (*P* < 0.05). Myofibrillar protein synthesis rates averaged 0.041 ± 0.010, 0.036 ± 0.010, and 0.032 ± 0.007%·h^−1^ in WHEY, COLL and PLA, respectively, with only WHEY resulting in higher rates when compared with PLA (*P* < 0.05). Muscle connective protein synthesis rates averaged 0.072 ± 0.019, 0.068 ± 0.017, and 0.058 ± 0.018%·h^−1^ in WHEY, COLL, and PLA, respectively, with no significant differences between groups (*P* = 0.09).

**Conclusions:**

Ingestion of whey protein during recovery from exercise increases myofibrillar protein synthesis rates. Neither collagen nor whey protein ingestion further increased muscle connective protein synthesis rates during the early stages of postexercise recovery in both male and female recreational athletes.

Acute exercise strongly increases muscle protein synthesis rates, thereby facilitating the skeletal muscle adaptive response to prolonged exercise training (1,2). Protein ingestion has been shown to further augment postexercise muscle protein synthesis rates, leading to net muscle protein accretion during recovery from exercise (3,4). The capacity of protein ingestion to stimulate postexercise muscle protein synthesis rates has been shown to depend on both the quality and amount of protein ingested (5–8). Ingestion of 20 g of a high-quality protein source, such as egg or whey protein, have been reported to maximize postexercise muscle protein synthesis rates in healthy adults (6,9). Consequently, athletes are typically advised to consume 20 to 25 g of high-quality protein during recovery from an exercise bout to support muscle conditioning and, as such, optimize exercise training adaptations.

Besides the force-generating contractile apparatus (i.e., myofibrillar proteins), skeletal muscle tissue also contains a complex network of connective proteins responsible for transferring these forces along the muscle (10). More specifically, up to 80% of the contractile force is transferred laterally through the intracellular and extracellular connective protein network before reaching the tendon to facilitate joint movement (11,12). The quality of the muscle connective protein network is a key factor determining the capacity for muscle to generate force. It should not be surprising that, along with the myofibrillar proteins, the muscle connective proteins are also under constant remodeling. Furthermore, connective protein synthesis rates increase in response to both an acute bout of exercise (13–18), as well as longer-term period of exercise training (19).

It has been well established that protein ingestion during recovery from exercise increases myofibrillar protein synthesis rates (5,9). However, it remains unclear whether muscle connective protein synthesis rates are responsive to protein ingestion during postexercise recovery. We recently showed that the ingestion of up to 40 g casein protein does not further augment postexercise muscle connective protein synthesis rates (16,17). This seems to be in line with some (13,20–23) but not all (24) studies investigating the effect of protein ingestion on muscle connective protein synthesis rates. All of these studies administered their subjects either dairy protein or a select set of essential amino acids. It has been suggested that these protein sources may not provide sufficient amino acid precursors to support a further increase in postexercise muscle connective protein synthesis rates (25,26). With collagen being the major structural protein in connective tissue (11), the most abundant amino acids found in connective tissue structures are glycine and proline (constituting ~25 and 12%, respectively (27)). It has been argued that, in contrast to dairy protein, ingestion of dietary collagen may be more effective in stimulating muscle connective protein synthesis rates, due to its high glycine and proline contents (25,28). In fact, Shaw et al. (29) reported that plasma samples obtained after collagen ingestion *in vivo* in humans, stimulate *in vitro* collagen synthesis when applied on engineered ligaments. Although it has been shown that collagen protein ingestion does not stimulate myofibrillar protein synthesis rates (19,30), no study to date has assessed the impact of collagen protein ingestion on postexercise myofibrillar and muscle connective protein synthesis rates.

We hypothesized that, in contrast to whey protein, collagen protein ingestion increases postexercise muscle connective protein synthesis rates. Furthermore, we hypothesized that, in contrast to collagen protein, whey protein ingestion increases postexercise myofibrillar protein synthesis rates. To test our hypotheses, we selected 45 healthy young men and women to ingest 30 g whey protein, 30 g collagen protein, or a noncaloric placebo after a single bout of resistance exercise. Primed, continuous intravenous l-[ring-^13^C_6_]-phenylalanine infusions were applied with blood and muscle tissue samples collected frequently to assess postexercise myofibrillar as well as muscle connective protein synthesis rates during recovery from exercise in male and female recreational athletes.

## METHODS

### Subjects

A total of 45 healthy, recreationally active men and women (age, 25 ± 4 yr; body mass index [BMI], 24.1 ± 2.0 kg·m^−2^) with prior experience with resistance exercise training including free weights (>6 months, 3× per week) volunteered to participate in this parallel-group, double-blind, randomized controlled trial. Subjects’ characteristics are presented in Table 1. After pretesting, subjects were randomly assigned to one of three groups consuming either 30 g whey protein (WHEY), 30 g collagen protein (COLL) or a noncaloric placebo (PLA). All subjects were informed of the nature and possible risks of the experimental procedures before their written informed consent was obtained. This study was approved by the Medical Ethical Committee of the Maastricht University Medical Centre+ and conforms to the principles outlined in the declaration of Helsinki for use of human subjects and tissue. The trial was registered at the Netherlands Trial Register (NL8814) and was conducted between February 2020 and December 2020 at Maastricht University Medical Centre+, Maastricht, The Netherlands. Clinical Trial Center Maastricht independently monitored the study.

**TABLE 1:** Subjects’ characteristics and average 2-d dietary intake before the experimental period.

	WHEY (*n* = 15)	COLL (*n* = 15)	PLA (*n* = 15)
Age (yr)	25 ± 4	27 ± 5	24 ± 4
Sex (m/f)	10/5	10/5	10/5
Height (m)	1.76 ± 0.09	1.80 ± 0.10	1.79 ± 0.11
Body mass (kg)	73.5 ± 11.9	80.5 ± 13.4	78.0 ± 11.3
BMI (kg·m^−2^)	23.5 ± 2.4	24.7 ± 2.2	24.2 ± 1.4
Lean body mass (kg)	59.2 ± 10.1	64.7 ± 11.7	61.2 ± 11.9
Body fat (%)	19.6 ± 5.2	19.5 ± 6.9	22.1 ± 6.3
*Rectus femoris* CSA (mm^2^)	666 ± 175	696 ± 179	652 ± 205
1RM barbell squat (kg)	111 ± 44	115 ± 25	118 ± 37
Energy (MJ·d^−1^)	9.51 ± 2.25	9.83 ± 2.96	9.68 ± 3.09
Carbohydrate (g)	246 ± 75	251 ± 97	250 ± 99
Fat (g)	88 ± 23	85 ± 26	88 ± 32
Protein (g)	100 ± 37	131 ± 48	111 ± 50
Protein (g·kg^−1^ body mass per day)	1.3 ± 0.4	1.6 ± 0.5	1.4 ± 0.5
Vitamin C (mg·d^−1^)	137 ± 49	132 ± 48	146 ± 48

Values represent means ± SD. Data were analyzed with a one-way ANOVA. There were no differences between treatments.

CSA, cross-sectional area.

### Pretesting

Participants (age 18–35 yr with a BMI > 18.5 and <30.0 kg·m^−2^) underwent an initial screening session to assess height, body weight, body composition (BIA, BioScan 920, Maltron International Ltd, UK), and the one repetition maximum (1RM) of the barbell squat exercise. The 1RM was estimated using the multiple repetitions testing procedure and calculated using the Brzycki equation (31). Participants were deemed healthy based on their responses to a medical questionnaire and were excluded from participation if smoking, using medication that affected protein metabolism, having any musculoskeletal diseases, or intolerant to the investigated protein products. The pretesting and experimental trials were separated by at least 5 d.

### Diet and physical activity

All participants refrained from strenuous physical activity and alcohol consumption and filled out food intake and physical activity questionnaires for 2 d before the experimental trial. Habitual dietary intake data were analyzed using online software available from the Dutch Health Council *(Mijn Eetmeter:* https://mijn.voedingscentrum.nl/nl/eetmeter/) and are presented in Table 1. Participants consumed the same standardized meal before 21.00 on the evening before the experimental trial. This prepackaged standardized meal provided 1.71 MJ, with 55% of the energy from carbohydrate, 30% energy from fat, and 15% energy from protein. Thereafter, participants remained fasted until the experimental test day.

### Study design

Participants performed barbell squatting exercise before consuming a randomly assigned beverage (500 mL) containing either 30 g whey protein (WHEY), 30 g collagen protein (COLL) or placebo (PLA). Nutri® Whey Isolate (FrieslandCampina, The Netherlands) and collagen protein hydrolysate (ATRO ProVita GmbH, Germany) were dissolved in water after which beverages were flavored with vanilla flavoring (Dr. Oetker, The Netherlands). The noncaloric placebo (PLA) was flavored water. Amino acid profiles of the protein beverages are shown in Table 2. Neither the whey nor the collagen protein supplement contained additional vitamin C. Randomization was performed using a computerized list randomizer (http://www.randomization.com/). Participants were sequentially allocated to the treatment groups, stratified by sex, by an independent researcher, according to the randomized list. The study beverages were prepared by an independent researcher in nontransparent plastic containers and had a similar taste and smell. All women were tested in the same phase of the menstrual cycle (within the first 7 d of the follicular phase).

**TABLE 2:** Amino acid profiles of the test beverages.

Amino Acids	WHEY	COLL	PLA
Alanine (g)	1.44	2.50	0
Arginine (g)	0.56	2.12	0
Aspartic acid (g)	3.30	1.69	0
Cysteine (g)	0.67	0	0
Glutamic acid (g)	5.41	2.97	0
Glycine (g)	0.44	6.46	0
Histidine (g)	0.44	0.29	0
Hydroxylysine (g)	0	0.47	0
Hydroxyproline (g)	0	3.46	0
Isoleucine (g)	1.97	0.41	0
Leucine (g)	3.00	0.79	0
Lysine (g)	2.78	1.05	0
Methionine (g)	0.64	0.26	0
Phenylalanine (g)	0.83	0.61	0
Proline (g)	1.80	3.70	0
Serine (g)	1.39	0.93	0
Threonine (g)	2.08	0.52	0
Tryptophan (g)	0.89	0	0
Tyrosine (g)	1.75	0.23	0
Valine (g)	0.50	0.70	0
ΣNEAA (g)	16.76	24.53	0
ΣEAA (g)	13.13	4.63	0
ΣAA (g)	29.89	29.16	0

ΣNEAA, sum total nonessential amino acids; ΣEAA, sum total essential amino acids; ΣAA, sum total amino acids.

### Experimental protocol

For a schematic representation of the primed continuous infusion protocol, see Supplemental Figure 1 (Supplemental Digital Content, http://links.lww.com/MSS/C864). At ∼07:45 am, participants arrived at the laboratory in an overnight fasted state. A catheter was inserted into an antecubital vein for stable isotope amino acid infusion. Subsequently, a second catheter was inserted into a dorsal hand vein of the contralateral arm for arterialized venous blood sampling. To obtain arterialized blood samples, the hand was placed in a hot box (60°C) for 10 min before blood sample collection. After taking a baseline blood sample (*t* = −180 min), the plasma phenylalanine pool was primed with a single intravenous dose (priming dose) of l-[ring-^13^C_6_]-phenylalanine (3.15 μmol·kg^−1^) and l-[3,5-^2^H_2_]-tyrosine (1.20 μmol·kg^−1^). After priming, a continuous intravenous infusion of l-[ring-^13^C_6_]-phenylalanine (0.070 μmol·kg^−1^·min^−1^) and l-[3,5-^2^H_2_]-tyrosine (0.027 μmol·kg^−1^·min^−1^) was initiated and maintained using a calibrated IVAC 598 pump. Thereafter, *rectus femoris* cross-sectional area was determined by ultrasound (MyLab Gamma, Esaote, Italy). After resting in a supine position, a second and third arterialized blood sample were drawn (*t* = −120 min; *t* = −60 min). After resting for another 25 min, participants initiated (*t* = −35 min) the resistance exercise intervention (described below). Immediately after the exercise intervention (*t* = 0 min), an arterialized blood sample was obtained, and a muscle biopsy sample was collected from the *vastus lateralis* muscle of a randomly selected leg. Subsequently, participants received a 500-mL beverage corresponding to their randomly assigned treatment allocation (WHEY, *n* = 15; COLL, *n* = 15; PLA, *n* = 15). Sequential arterialized blood samples were collected at *t* = 30, 60, 90, 120, 180, 240, and 300 min throughout the postprandial period. The second muscle biopsy sample was collected at t = 300 min to determine postprandial myofibrillar and muscle connective protein synthesis rates (*t* = 0–300 min). When the experimental protocol was complete, the cannulas were removed, and participants ate and were monitored for ∼30 min before leaving the laboratory.

### Blood and muscle tissue sampling

Blood samples were collected into EDTA-containing tubes and centrifuged at 1000*g* for 15 min at 4°C. Aliquots of plasma were frozen in liquid nitrogen and stored at −80°C. Biopsy samples were collected using a 5-mm Bergström needle custom-adapted for manual suction. Samples were obtained from separate incisions from the middle region of the *vastus lateralis*, ∼15 cm above the patella and ∼3 cm below entry through the fascia, under 1% xylocaine local anesthesia with adrenaline (1:100,000). Muscle samples were freed from any visible nonmuscle material, immediately frozen in liquid nitrogen, and stored at −80°C until further processing.

### Resistance exercise session

All participants followed the same exercise protocol that consisted of a 5-min warm-up on a cycle ergometer followed by six sets of barbell squats. Barbell squats were performed with free weights from full knee extension to the point the thighs were parallel to the floor. The workload was set at 60% of 1RM with 15, 12, 10, 10, 8, and 8 repetitions per set. Resting periods of 2 min were allowed between all sets. Delayed onset of muscle soreness (DOMS) of the legs was self-reported by the subjects 24 and 48 h after cessation of the training session using a seven-point Likert scale of muscle soreness with a score of 0 indicating complete absence of soreness and 6 indicating severe pain that limits the ability to move (32).

### Plasma analysis

Plasma glucose and insulin concentrations were analyzed using commercially available kits (GLUC3, Roche, Ref: 05168791 190, and Immunologic, Roche, Ref: 12017547 122, respectively). Quantification of plasma amino acid concentrations was performed using ultra-performance liquid chromatograph mass spectrometry (UPLC-MS; ACQUITY UPLC H-Class with QDa; Waters, Saint-Quentin, France). Blood plasma (50 μL) was deproteinized using 100 μL of 10% SSA with 50 μM of MSK-A2 internal standard (Cambridge Isotope Laboratories, Andover, MA). Subsequently, 50 μL of ultra-pure demineralized water was added, and samples were centrifuged (15 min at 14,000 RPM). After centrifugation, 10 μL of supernatant was added to 70 μL of Borate reaction buffer (Waters, Saint-Quentin, France). In addition, 20 μL of AccQ-Tag derivatizing reagent solution (Waters, Saint-Quentin, France) was added after which the solution was heated to 55°C for 10 min. An aliquot of 1 μL was injected and measured using UPLC-MS. Plasma amino acid enrichments were determined by gas chromatography-mass spectrometry analysis (GC-MS; Agilent 7890A GC/5975C; MSD, Wilmington, DE). The plasma l-[ring-^13^C_6_]-phenylalanine enrichments were determined using selective ion monitoring at m/z 336, 337, and 341 for unlabeled and labeled (^13^C) phenylalanine. Standard regression curves were applied from a series of known standard enrichment values against the measured values to assess the linearity of the mass spectrometer and to account for any isotope fractionation.

### Muscle tissue analysis

Muscle connective and myofibrillar protein-enriched fractions were isolated from ~100 mg of wet muscle tissue by hand homogenizing on ice using a pestle in a standard extraction buffer (10 μL·mg^−1^). The samples were spun for 15 min at 700*g* and 4°C. The supernatant was transferred to a separate tube for Western blot analysis. The pellet was washed with 400 μL of extraction buffer before vortexing and centrifugation at 700*g* and 4°C for 10 min. The supernatant was removed, and the pellet was washed with 500 μL ddH_2_O before vortexing and centrifugation at 700*g* and 4°C for 10 min. The supernatant was removed, and 1 mL of homogenization buffer was added, and the material was suspended by vortexing before transferring into microtubes containing 1.4 mm ceramic beads and Lysing Matrix D (MP Biomedicals, Irvine, CA). The microtubes were vigorously shaken four times for 45 s at 5.5 m·s^−1^ (FastPrep-24 5G; MP Biomedicals) to mechanically lyse the protein network. Samples were then left to rest at 4°C for 3 h before centrifugation at 700*g* and 4°C for 20 min, discarding the supernatant and adding 1 mL of homogenization buffer. The microtubes were shaken for 40 s at 5.5 m·s^−1^ before centrifugation at 700*g* and 4°C for 20 min. The supernatant was discarded, and 1 mL of KCl buffer was added to the pellets before being vortexed and left to rest overnight at 4°C. The next morning, samples were vortexed and centrifuged at 1600*g* for 20 min at 4°C.

For the myofibrillar isolation the supernatant was transferred to a separate tube. Then, 3.4 mL EtOH 100% was added, samples were vortexed, left for 2 h at 4°C and then centrifuged at 1600*g*, for 20 min at 4°C. The supernatant was discarded and EtOH 70% was added to the pellet, vortexed and centrifuged again at 1600*g*, for 20 min at 4°C. The supernatant was again discarded and the remaining pellet was suspended in 2 mL of 6 M HCl in glass screw-cap tubes and left to hydrolyze overnight at 110°C.

For the connective protein isolation, the pellet, containing both immature and mature connective proteins, was mixed with 1 mL KCl buffer and left for 2 h at 4°C. The samples were vortexed and centrifuged at 1600*g* for 20 min at 4°C, and the supernatant was discarded. To the pellet 1 mL ddH2O were added, vortexed, left for 2 h at 4°C and then centrifuged at 1600*g*, for 20 min at 4°C. The supernatant was removed and the remaining pellet was suspended in 1 mL of 6M HCl in glass screw-cap tubes and left to hydrolyze overnight at 110°C.

After hydrolyzation, the free amino acids were then dissolved in 25% acetic acid solution, passed over cation exchange AG 50W-X8 resin columns (mesh size, 100–200; ionic form, hydrogen; Bio-Rad Laboratories, Hercules, CA), washed five times with water and finally eluted with 2M NH4OH. To determine myofibrillar and connective protein l-[ring-^13^C_6_]-phenylalanine enrichments by GC-IRMS analysis, the purified amino acids were converted into N-ethoxycarbonyl ethyl ester derivatives with ethyl chloroformate. The samples were measured using a gas chromatography-isotope ratio mass spectrometer (Finnigan MAT 252; Thermo Fisher Scientific, Bremen, Germany) equipped with an Ultra I GC-column (no. 19091A-112; Hewlett-Packard, Palo Alto, CA) and combustion interface II (GC-C-IRMS). Ion masses 44, 45, and 46 were monitored for ^13^C phenylalanine. By establishing the relationship between the enrichment of a series of l-[ring-^13^C_6_]-phenylalanine, standards of variable enrichment and the enrichment of the N(O,S)-ethoxycarbonyl ethyl esters of these standards, the muscle–protein-bound enrichment of phenylalanine was determined.

### Whole body collagen turnover markers

Procollagen type I N propeptide (P1NP) and carboxy-terminal crosslinking telopeptide of type I collagen (CTX-I) were selected as markers of bone/collagen formation and bone/collagen resorption, respectively, in line with the recommendation by The International Osteoporosis Foundation (IOF) and International Federation of Clinical Chemistry (IFCC) (33). P1NP and CTX-I were measured at four time points from plasma samples (*t* = −120; *t* = 0; *t* = 120; *t* = 300 min). Intact PINP and CTX-I were measured using chemiluminescent immunometric assays on the IDS-iSYS instrument (Immunodiagnostic Systems, PLC), by the Central Diagnostic Laboratory at the Maastricht University Medical Centre (The Netherlands).

### Calculations

The fractional synthetic rate (FSR) of myofibrillar and muscle connective protein was calculated by dividing the increment in myofibrillar and connective protein enrichment by weighted mean precursor (plasma) amino acid tracer enrichment. Consequently, myofibrillar and connective protein FSR were calculated as follows:


$$\mathrm{FSR}\;\left(\%\cdot h^{-1}\right)=\left(\frac{E_{m2}-E_{m1}}{E_{\text{precursor}}\times t}\right)\times 100\%\;\left[1\right]$$

*E*_m1_ and *E*_m2_ represent protein-bound l-[ring-^13^C_6_]-phenylalanine, *E*_precursor_ represents the average plasma free l-[ring-^13^C_6_]-phenylalanine enrichment during the tracer incorporation period, and t indicates the time interval (h) between biopsies. Basal myofibrillar and muscle connective protein FSR were calculated based on plasma albumin enrichment at *t* = −180 min and myofibrillar and muscle connective enrichments at *t* = 0 min (34,35).

### Statistical analysis

A power calculation was performed with differences in postprandial muscle FSR between the groups as primary outcome measure. A minimum sample size of 14 participants per treatment, was calculated using a power of 80%, a significance level of 0.01667 (adjusted from 0.05 for multiple comparisons), an SD of 0.006%·h^−1^, and a difference in FSR of 0.008%·h^−1^ between treatments (or ~20% when expressed as a relative difference). To account for potential drop-outs we included one extra participant per treatment for a total of 15 participants per group. All data in text are expressed as mean ± SD. Baseline characteristics and dietary intake between groups were compared using a one-way ANOVA. The trapezoidal rule adjusted to baseline concentration (*t* = 0) was applied to calculate the incremental area under curve (iAUC) of the amino acid concentrations. Time-dependent variables (i.e., DOMS, plasma glucose, insulin, amino acid concentrations, and tracer enrichments) were analyzed by a mixed model ANOVA with time as a within-subjects factor and treatment group as a between-subjects factor. The analysis was carried out for the period starting at the time of protein or placebo ingestion (*t* = 0 min) until the end of the experimental trial (*t* = 300 min). In case of a significant interaction effect, individual time points were analyzed using a one-way ANOVA with the time points as the dependent variable and treatment as the independent variable. Non–time-dependent variables (i.e., postexercise myofibrillar and muscle connective protein FSR and iAUC) were compared between treatment groups using a one-way ANOVA. A secondary statistical analysis has been performed on myofibrillar and muscle connective protein FSR in a time-dependent manner with basal FSR and postprandial FSR using a two-factor repeated-measures ANOVA with time as a within-subjects factor and treatment group as a between-subjects factor. Bonferroni-corrected *post hoc* comparisons were performed where appropriate. Statistical significance was set at *P* < 0.05. All calculations were performed using SPSS 24.0 (SPSS Inc., Chicago, IL).

## RESULTS

### Participants’ characteristics and habitual dietary intake

There were no significant differences in the participants’ characteristics between the three treatment groups (Table 1). In line, there were no differences in habitual dietary intake between treatment groups (Table 1). Dietary vitamin C intake of the 2 d before the experimental test day averaged 137 ± 49, 132 ± 48, and 146 ± 48 mg·d^−1^ in WHEY, COLL, and PLA, respectively (*P* > 0.05). All participants ingested amounts of vitamin C above the Recommended Dietary Allowance of 90 mg·d^−1^ for men and 75 mg·d^−1^ for women (36).

### Plasma glucose and insulin concentrations

**FIGURE 1:**
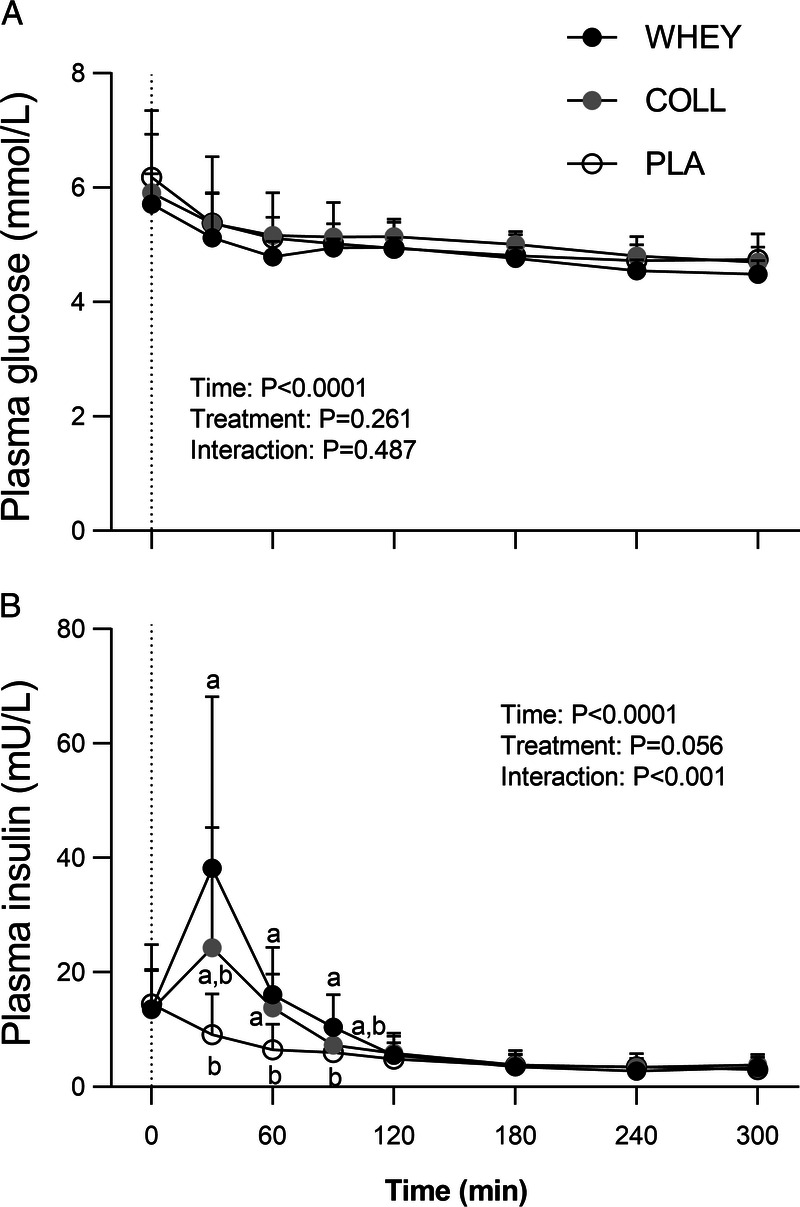
Postprandial plasma insulin (A) and glucose (B) concentrations following whey, collagen, or placebo ingestion during recovery from a single bout of resistance exercise (*t* = 0–300 min). The *dotted line* represents the ingestion of the test drink. Values represent means ± SD, *n* = 15 per group. Data were analyzed by a two-factor repeated-measures ANOVA. Bonferroni *post hoc* testing was used to detect differences between groups. Treatments without a common letter differ, *P* < 0.05.

Plasma glucose concentrations declined over time (*P* < 0.05), with no differences between treatments (Fig. 1A). Both whey and collagen protein ingestion resulted in significant increases in circulating insulin concentrations, with values exceeding those observed in the placebo treatment at *t* = 30–90 min and *t* = 60 min in the whey and collagen treatments, respectively (time–treatment group interaction*, P* < 0.05; Fig. 1B).

### Plasma collagen turnover markers

Plasma P1NP values increased over time (*P* < 0.05), with differences observed between *t* = 120 and *t* = 300 min in all groups (*P* < 0.05). A significant time–treatment group interaction was observed for plasma CTX-I concentrations (*P* < 0.001), with differences observed at *t* = 120 min in both WHEY and COLL versus PLA (*P* < 0.05), with no differences between WHEY and COLL. Results for P1NP and CTX-I are shown in Supplemental Figure 2 (Supplemental Digital Content, http://links.lww.com/MSS/C864).

### Plasma amino acid concentrations

**FIGURE 2:**
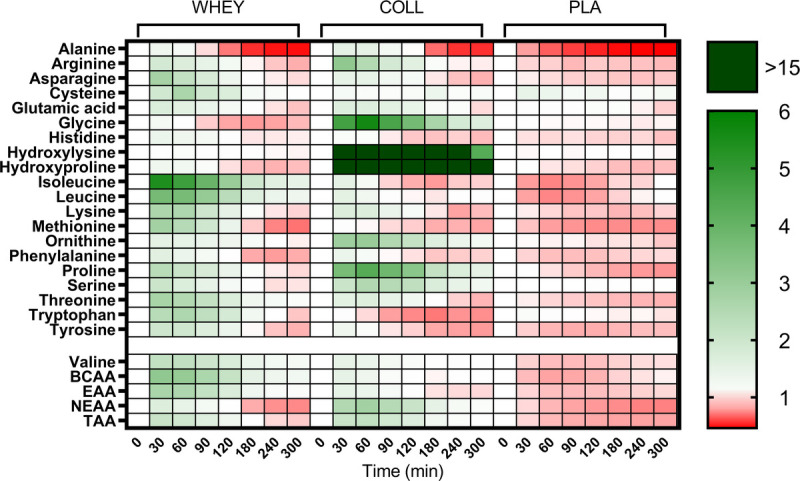
Heat map of fold changes in plasma amino acid concentrations during the experimental test day with whey, collagen, or placebo ingestion during recovery from a single bout of resistance exercise, *n* = 15 per group. TAA, total amino acids; EAA, essential amino acids; BCAA, branched-chain amino acids. For hydroxyproline values under the detection limit were set to 0.

Results for all measured amino acids are visualized in a heat map showing the fold-change in plasma amino acid concentration following test drink ingestion when compared with baseline (Fig. 2).

**FIGURE 3:**
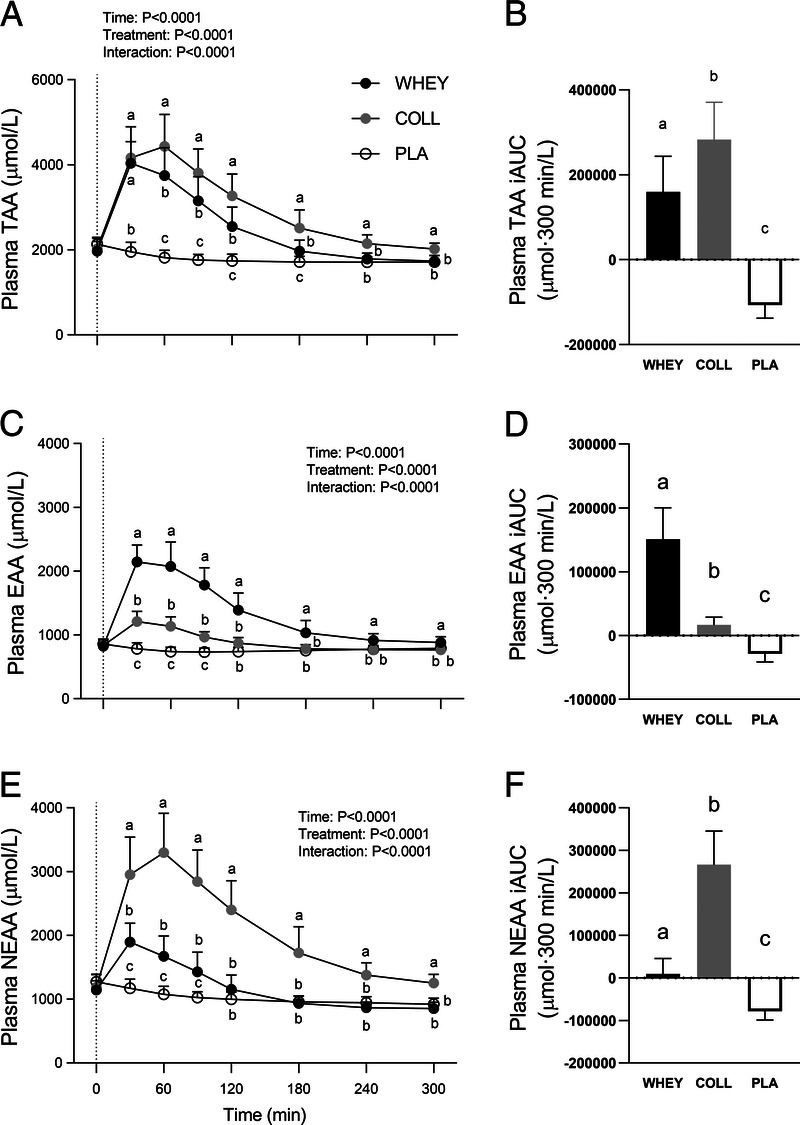
Postprandial plasma amino acid concentrations following whey, collagen, or placebo ingestion during recovery from a single bout of resistance exercise (*t* = 0–300 min) in the left column and iAUC for the post prandial time frame in the right column. Data are displayed for TAA (A, B), EAA (C, D) and NEAA (E, F). The dotted line within the left column graphs represents the ingestion of the test drink. Values represent means ± SD, *n* = 15 per group. Data for plasma amino acid concentrations were analyzed by a two-factor repeated-measures ANOVA. Data for iAUC were analyzed by a one-way ANOVA. Bonferroni *post hoc* testing was used to detect differences between groups. Treatments without a common letter differ, *P* < 0.05.

**FIGURE 4:**
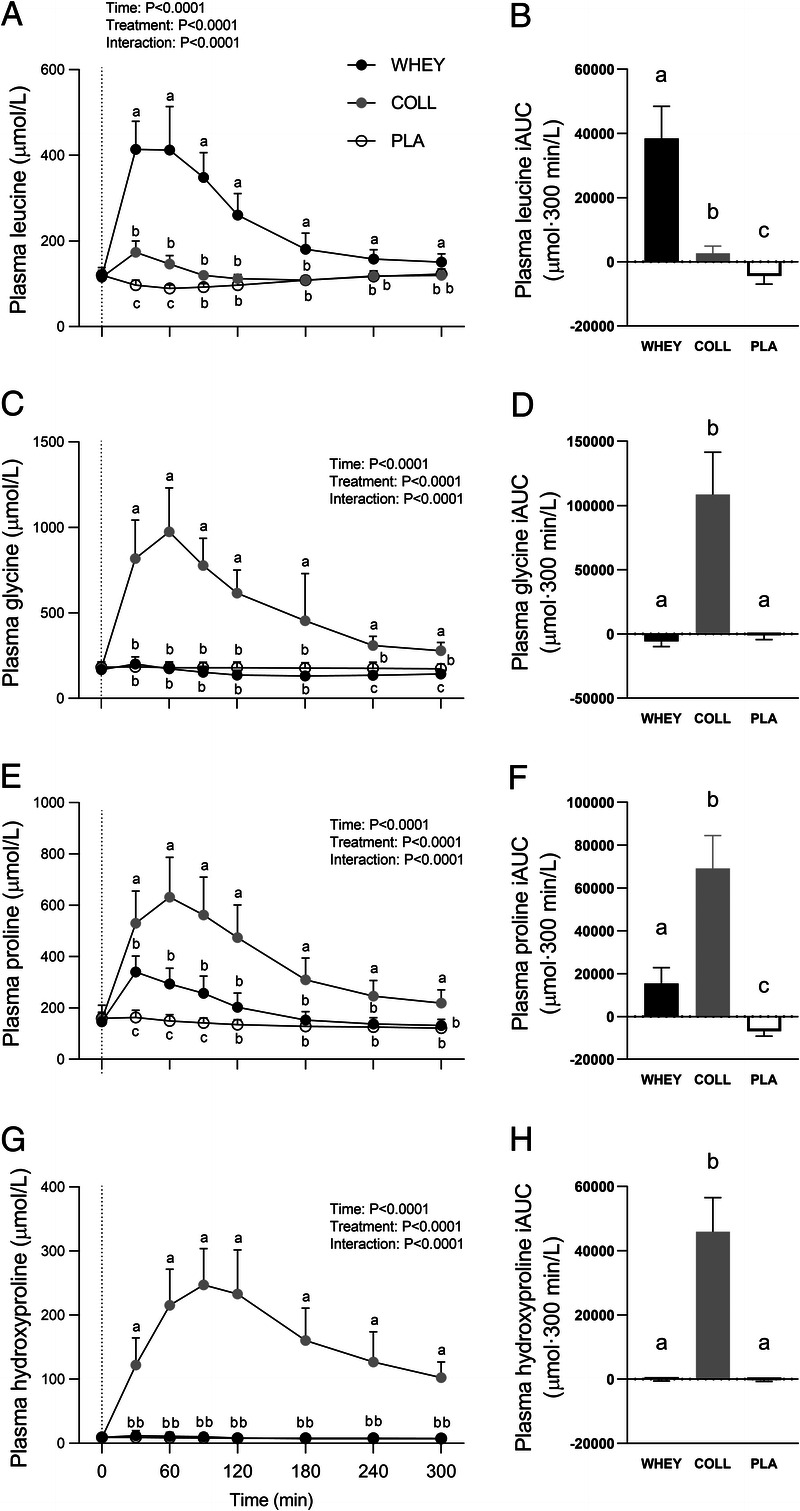
Postprandial plasma leucine, glycine, proline, and hydroxyproline concentrations following whey, collagen, or placebo ingestion during recovery from a single bout of resistance exercise (*t* = 0–300 min) in the left column and incremental area under the curve (iAUC) for the post prandial time frame in the right column. Data are displayed for leucine (A, B), glycine (C, D), proline (E, F), and hydroxyproline (G, H). The *dotted line* within the left column graphs represents the ingestion of the test-drink. Values represent means ± SD, *n* = 15 per group. Data for plasma amino acid concentrations were analyzed by a two-factor repeated-measures ANOVA. Data for iAUC were analyzed by a one-way ANOVA. Bonferroni *post hoc* testing was used to detect differences between groups. Treatments without a common letter differ, *P* < 0.05.

Plasma amino acid concentrations are shown in Figures 3 and 4. Significant time–treatment group interactions were observed for all plasma amino acid concentrations (all *P* < 0.001). Protein ingestion increased plasma total (TAA), essential (EAA), non-essential (NEAA), leucine, and proline concentrations. Collagen protein ingestion further increased TAA concentrations from *t* = 60 to 300 min, and plasma NEAA and proline concentrations from *t* = 30 to 300 min compared with whey protein ingestion (*P* < 0.05). These differences are reflected in the iAUC values with plasma TAA, NEAA, and proline concentrations over the entire postprandial time frame being the highest in COLL, followed by WHEY, and lowest in PLA (*P* < 0.05). Whey protein further increased plasma EAA and leucine concentrations from *t* = 30 to 300 min compared with collagen protein ingestions (*P* < 0.05). Those results are reflected in the iAUC with plasma EAA and leucine concentrations over the entire postprandial time frame being the highest in WHEY, followed by COLL, and lowest in PLA (*P* < 0.05). Plasma glycine and hydroxyproline concentrations were increased after the ingestion of collagen protein only for the entire postprandial time frame (*P* < 0.05). The ingestion of whey protein decreased plasma glycine concentrations at *t* = 240 and 300 min compared with PLA. Results of the iAUC over the whole postprandial time frame showed the highest plasma glycine and hydroxyproline concentrations in COLL, with no differences between WHEY and PLA (*P* < 0.05).

### Stable isotope tracer analyses

**FIGURE 5:**
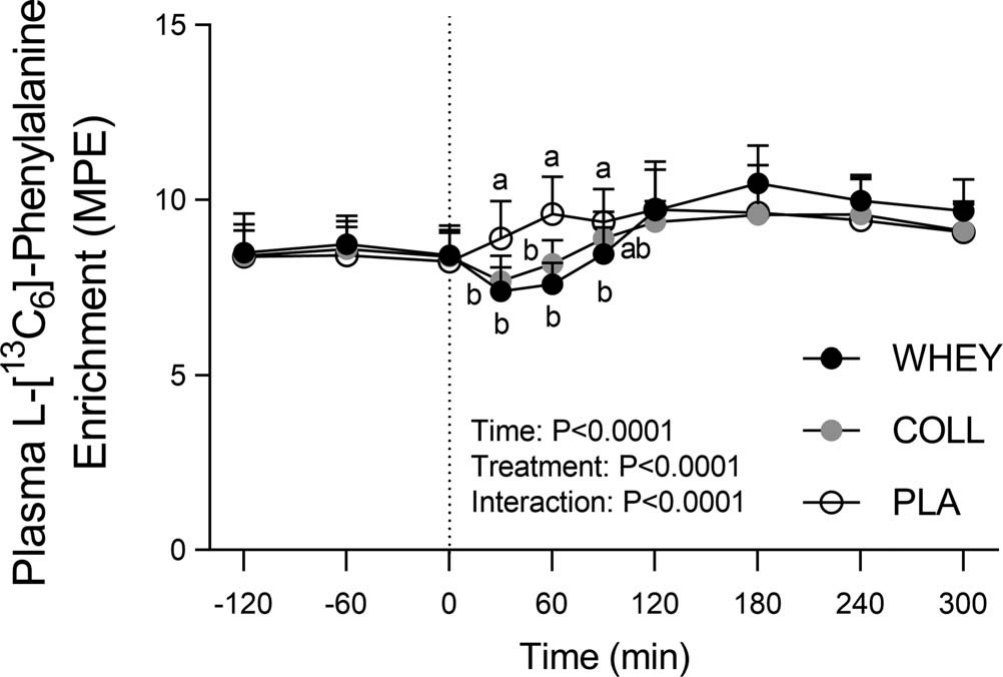
Plasma l-[ring-^13^C_6_]-phenylalanine enrichments (MPE) before (*t* = −120-0 min) and after whey, collagen, or placebo ingestion during recovery from a single bout of resistance exercise (*t* = 0–300 min). The *dotted line* represents the ingestion of the test drink. Values represent means ± SD, *n* = 15 per group. Data were analyzed by a two-factor repeated-measures ANOVA. Bonferroni *post hoc* testing was used to detect differences between groups. Treatments without a common letter differ, *P* < 0.05.

Analysis of plasma l-[ring-^13^C_6_]-phenylalanine enrichments reveled a significant time–treatment group interaction effect (*P* < 0.05; Fig. 5). During the early postprandial phase (*t* = 30 to 60 min), plasma l-[ring-^13^C_6_]-phenylalanine enrichments were lower in WHEY and COLL compared with PLA (*P* < 0.05). However, weighted plasma l-[ring-^13^C_6_]-phenylalanine enrichments over the entire 5 h postprandial period did not differ between groups (*P* > 0.05).

### Myofibrillar protein synthesis

**FIGURE 6:**
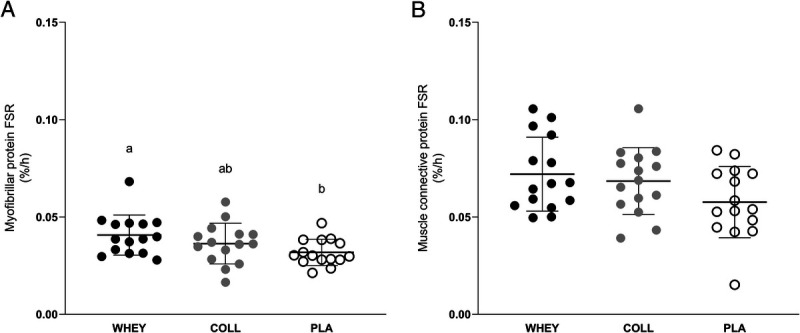
Fractional myofibrillar (A) and muscle connective (B) protein synthesis rates (%^.^h^−1^) after whey, collagen, or placebo ingestion during recovery from a single bout of resistance exercise (*t* = 0–300 min). Values represent means ± SD, and dots represent individual values, *n* = 15 per group. Data were analyzed using one-factor ANOVAs. Bonferroni *post hoc* testing was used to detect differences between groups. Treatments without a common letter differ, *P* < 0.05. *Post hoc* testing for myofibrillar FSR: WHEY vs COLL *P* = 0.606, WHEY vs PLA *P* = 0.037, COLL vs PLA *P* = 0.581.

Postabsorptive myofibrillar protein synthesis rates calculated using the single biopsy approach averaged 0.009 ± 0.007, 0.009 ± 0.007, and 0.008 ± 0.008%·h^−1^ in the WHEY, COLL, and PLA groups, respectively, with no differences between treatment groups (main effect *P* > 0.05). Postexercise myofibrillar protein synthesis rates were significantly higher compared with postabsorptive rates at 0.040 ± 0.010, 0.036 ± 0.010, and 0.032 ± 0.007%·h^−1^ in the WHEY, COLL, and PLA groups, respectively (time effect *P* < 0.05; Fig. 6A), with myofibrillar protein synthesis rates in WHEY being higher compared with PLA (*P* < 0.05). Postexercise myofibrillar protein synthesis rates in COLL did not differ from PLA or WHEY (*P* > 0.05 for both comparisons).

### Muscle connective protein synthesis

Postabsorptive muscle connective protein synthesis rates calculated using the single biopsy approach averaged 0.029 ± 0.012, 0.034 ± 0.012, and 0.028 ± 0.014%·h^−1^ in the WHEY, COLL, and PLA groups, respectively, with no differences between treatments (*P* > 0.05). Postexercise muscle connective protein synthesis rates were significantly higher compared with postabsorptive rates at 0.072 ± 0.019, 0.068 ± 0.017, and 0.058 ± 0.018%·h^−1^ in the WHEY, COLL, and PLA groups, respectively (time effect *P* < 0.05; Fig. 6B), with no differences between groups (*P* = 0.09).

### Delayed onset muscle soreness

Delayed onset of muscle soreness of the legs on a seven-point Likert scale averaged 2.6 ± 1.3, 2.3 ± 1.7, and 2.7 ± 1.2 after 24 h and 2.6 ± 1.7, 2.1 ± 1.7, and 2.3 ± 1.6 after 48 h of postexercise recovery in the WHEY, COLL, and PLA group, respectively, with no differences between treatments (*P* > 0.05 for both time points).

## DISCUSSION

In the present study, we demonstrated that a single bout of barbell squat exercise increases both myofibrillar and muscle connective protein synthesis rates. Whey protein ingestion further increased postexercise myofibrillar protein synthesis rates when compared with the placebo condition, but failed to increase muscle connective protein synthesis rates. Ingestion of collagen protein did not increase myofibrillar or muscle connective protein synthesis rates during exercise recovery when compared with the placebo condition.

Resistance exercise has been shown to strongly increase both myofibrillar (1,37) and muscle connective (13,15,16) protein synthesis rates *in vivo* in humans. In the present study, we confirm these findings by reporting a substantial increase in myofibrillar (postabsorptive FSR of 0.008 ± 0.008%·h^−1^ to postexercise FSR of 0.032 ± 0.007%·h^−1^) and muscle connective protein synthesis rates (postabsorptive FSR of 0.028 ± 0.014%·h^−1^ to postexercise FSR of 0.058 ± 0.018%·h^−1^) during recovery from exercise when compared with basal, resting values in the placebo group. Muscle connective protein synthesis rates during recovery from exercise seem to be higher than previously reported by our laboratory (16) as well as others (13). Next to other factors, such as study design, incorporation times, and feeding strategies, this difference may be attributed to the type of exercise that was implemented in the current study design. Whereas previous studies typically applied isolated/machine-based leg exercise (such as leg extension and leg press) to assess the impact on myofibrillar and/or muscle connective protein synthesis rates (9,13,16,38), we applied a less isolated exercise task in the form of six sets of barbell squats. The barbell squat is an exercise found more often in athletes’ training programs, requires more balancing and muscle coordination and, as such, may have resulted in greater stimulation of muscle connective protein synthesis rates. In support, Wilk et al. (39) showed greater quadriceps muscle activation with a free weight barbell squat compared with leg press and leg extension exercise.

It has been well established that protein ingestion can further increase myofibrillar protein synthesis rates during recovery from exercise (6,9,40,41). The capacity to stimulate postexercise myofibrillar protein synthesis has been attributed to the postprandial rise in plasma amino acid concentrations, which stimulates anabolic signaling pathways and provides precursors for *de novo* protein synthesis (42,43). Here, we observed a rapid increase in plasma amino acid concentrations following both whey and collagen protein ingestion (Figs. 3 and 4). The postprandial rise in plasma amino acid concentrations differed substantially between the ingestion of whey versus collagen protein, reflecting their differences in amino acid composition (Table 2; Fig. 2). Whey protein ingestion resulted in a marked increase in plasma leucine concentrations which peaked 30 min after ingestion at ~400 μmol·L^−1^, while the ingestion of collagen protein resulted in peak leucine concentrations of merely ~170 μmol·L^−1^. On the other hand, collagen protein ingestion resulted in a more pronounced increase in plasma glycine, proline and hydroxyproline concentrations, reaching peak levels between 60 and 90 min after collagen ingestion. Though hydroxyproline has no function as a precursor for tissue protein synthesis, it provides us with proper insight in the rapid digestion of the ingested collagen protein and subsequent amino acid absorption (Figs. 2 and 4G, H). Our observations align with previous work by Alcock et al. (25) demonstrating that whereas dairy proteins are a superior source of leucine, collagen protein forms a superior source of glycine and proline. Although glycine and proline are nonessential amino acids, they represent important precursors for connective tissue remodeling, comprising ~37% of all amino acids within this protein fraction. It has been speculated that plasma glycine and proline availability may limit the postexercise increase in connective tissue protein synthesis rates. In line with the amino acid composition of both protein sources (Table 2), we observed large differences in the postprandial plasma essential and non-essential amino acid concentrations (Fig. 3). Postprandial plasma essential amino acid availability was ~9-fold higher following whey versus collagen protein ingestion, whereas the non-essential amino acid availability was ~30-fold higher following collagen compared with whey protein ingestion.

The postprandial rise in circulating amino acids following whey protein ingestion strongly increased postexercise myofibrillar protein synthesis rates when compared with the placebo condition (Fig. 6A). This finding is in line with previous studies reporting that ingestion of 20 g of high-quality protein strongly stimulates myofibrillar protein synthesis (9,41,44). In contrast, whey protein ingestion did not further increase muscle connective protein synthesis rates during recovery from exercise (Fig. 6B). This seems to be in line with most (13,20–23) but not all (24) studies investigating the impact of (dairy) protein ingestion on connective protein synthesis rates in muscle. Holm et al. suggested that the impact of postexercise protein ingestion, if any, may be more prominent during the latter stages of recovery from exercise. However, in recent studies, we failed to detect any impact of ingesting up to 40 g dairy protein before sleep on connective protein synthesis rates during an elongated overnight recovery period (7.5 h) in both young (16) and older (17) adults. Therefore, based on our previous work and the outcome of the present study, we conclude that ingestion of a high-quality dairy protein stimulates myofibrillar but not muscle connective protein synthesis rates during recovery from an acute bout of resistance exercise in recreational athletes.

Although it is evident that exercise stimulates muscle connective protein synthesis rates (13,15,16), thereby contributing to the conditioning of the connective protein network in muscle, thus far, it does not seem to be sensitive to a postprandial rise in plasma amino acid availability. However, it has been proposed that a stimulation of muscle connective protein synthesis rates may be dependent on the amino acid profile or the postprandial increase in amino acid availability (25,29). Much of the muscle connective protein network consists of collagen strands, which are abundant in glycine, proline and hydroxyproline (25%, 12%, and 13.5%, respectively). Therefore, it has been proposed that the postexercise increase in connective protein synthesis rates may be limited by the provision of ample glycine and proline precursors for collagen synthesis (16,29). In contrast to dairy protein, dietary collagen provides ample amounts of glycine and proline that could support a greater postexercise increase in muscle connective protein synthesis rates. Here, we assessed the impact of collagen protein ingestion on both myofibrillar protein and, for the first time, muscle connective protein synthesis rates (Fig. 6). In contrast to whey protein, collagen protein ingestion did not elevate myofibrillar protein synthesis rates. This may be attributed to the lower essential amino acid content of the 30 g collagen protein when compared with 30 g whey protein (~5 vs ~13 g essential amino acids, respectively) and/or more specifically to the differences in leucine content (0.8 vs 3 g leucine, respectively). In line with our results, Oikawa et al. (19) reported less of an increase in myofibrillar protein synthesis rates following ingestion of 30 g collagen versus 30 g whey protein ingestion, both at rest and during recovery from unilateral leg exercise. We extend on previous work by assessing, for the first time, whether collagen protein ingestion can stimulate muscle connective proteins synthesis rates during recovery from exercise. We hypothesized that dairy protein may not provide sufficient amounts of glycine and proline to maximize postexercise muscle connective protein synthesis rates (25), and, therefore, that dietary collagen protein may be the preferred protein source to stimulate muscle connective protein synthesis rates. In contrast to our hypothesis, despite the substantial postprandial rise in circulating glycine, proline and hydroxyproline concentrations, we failed to detect a further increase in muscle connective protein synthesis rates during the first 5 h of recovery from exercise (Fig. 6B). Although promising results of collagen supplementation have been reported for tendon (29), bone (45), and fat-free mass (46), muscle connective protein synthesis rates do not seem to be modulated by collagen ingestion during acute, postexercise recovery in recreationally active young athletes. Further research will be warranted to assess whether higher protein doses of collagen protein or blends of dairy and collagen protein will be effective to further increase muscle connective protein synthesis rates at rest and/or during recovery from different types of exercise.

Blood markers, such as P1NP and CTX-I, may be useful to assess the effect of exercise and nutritional interventions on whole body collagen synthesis and breakdown (47). Here, we observed a small increase in P1NP concentrations following resistance exercise. This finding is in accordance with a recent meta-analysis concluding that exercise in general results in a very small increase in P1NP concentrations. However, insufficient data were available to evaluate the P1NP response to an acute bout of resistance exercise specifically (48). CTX-I concentrations declined in all conditions in the present study until *t* = 120 min, in accordance with the well-known circadian rhythm of CTX-I (49). Also, in line with previous work (50), we observed that whole body collagen breakdown, as indicated by CTX-I, was inhibited by protein ingestion (for both WHEY and COLL), compared with the ingestion of water (PLA).

In the present study we show that both whey and collagen protein ingestion do not further increase muscle connective protein synthesis rates during the first few hours of recovery from exercise. Previously, we have shown that ingested protein provides precursors to support muscle connective protein synthesis rates, as we observed that (intrinsically labeled) casein protein derived amino acids are incorporated in *de novo* synthesized muscle connective protein (16,17). Though it is evident that dietary protein is required to support the conditioning of the muscle connective protein network, it seems clear that the early recovery phase from exercise does not require more amino acid precursors or specific dietary stimuli. The acute nature of the present study allows us to only speculate on the relevance of the amount and quality of dietary protein consumption (and collagen protein in particular) required to support muscle connective tissue conditioning when applied over a more prolonged intervention period. Furthermore, in the present study we assessed the impact of whey and collagen protein on connective protein within skeletal muscle tissue. Whether the reported findings also translate to other connective protein networks, such as connective proteins in ligaments, tendons, and bone remains to be elucidated. The latter structures contain a much higher collagen content (e.g., ~85% collagen content dry weight, 51) when compared with muscle tissue (~5%, 22). *De novo* synthesis of collagen-rich tissues would require greater amounts of glycine and proline to support tissue remodeling and may, therefore, be more dependent on collagen protein ingestion. Future studies will address these issues to elucidate whether there is a role for collagen protein in supporting the adaptive response of connective proteins to exercise in the various musculoskeletal tissues in both health and disease.

## CONCLUSIONS

In conclusion, resistance exercise increases both myofibrillar and muscle connective protein synthesis rates in male and female recreational athletes. Whey protein ingestion during recovery from exercise further increases myofibrillar but not muscle connective protein synthesis rates. Collagen protein ingestion during recovery from acute exercise does not increase myofibrillar nor muscle connective protein synthesis rates.
